# The application of a stress reduction intervention in patients with chronic diseases in Greece

**DOI:** 10.1192/j.eurpsy.2021.1921

**Published:** 2021-08-13

**Authors:** G. Lyrakos, M. Tsironi, E. Aslani, V. Spinaris

**Affiliations:** 1 Department Of Psychiatry, General Hospital of Nikaia “Ag. Panteleimon”, Nikaia, Greece; 2 Department Of Nursing, University of Peloponnese Faculty of Health Sciences, Tripoli, Greece; 3 2nd Department Of Pathology, General Hospital of Nikaia “Ag. Panteleimon”, Nikaia, Greece

**Keywords:** stress, relaxation techniques, stress intervention

## Abstract

**Introduction:**

This is the presentation of a postdoc research protocol under the supervision of University of Peloponnese, regarding the application of a program for stress reduction. Τhe intervention consisted of a psychoeducational session explaining the stress effect in the brain and the way that relaxation breathing is working, a training session on relaxation breathing and 2 more sessions, one with relaxation breathing and one with trigger points acupressure in the back of the patients.

**Objectives:**

The objective of the study was to test the effectiveness of relaxations techniques in patients with chronic diseases in a Greek General Hospital.

**Methods:**

Out of 335 participants, 153 were found to have high stress levels in the DASS scale and the stress VAS scale that was used for the intervention. 151 of them (49(32, 5%) males), agreed to participate in the intervention with relaxation breathing and 99 in the acupressure session. Diagnosis of the participants were: anxiety disorder (18.5%), Thalassaemia Major(31.1%), Crohn (26.5%), Kidney failure (9.9%) and major depression (13.9%). Analysis was performed with SPSS 24.

**Results:**

The results in DASS stress scale revealed that 54.3 % of the sample had very severe and 24.3% severe stress levels while 58.9% had very severe anxiety levels in DASS anxiety scale. Pair sample t test show statistically significant differences before and after the application of breathing relaxation t150=24.725 p=0.001, as well in the application of acupressure t98=15.901 p=0.001.

**Conclusions:**

According to the results of the present intervention, relaxation techniques can be very helpful as complimentary treatments for patients with chronic diseases

**Disclosure:**

No significant relationships.

